# Supplementary material for: Evaluation of Metabolic and Cardiovascular Risk Measured by Laboratory Biomarkers and Cardiopulmonary Exercise Test in Children and Adolescents Recovered from Brain Tumors: The CARMEP Study
Source: Cancers (Basel). 2024 Jan 11;16(2):324. doi: 10.3390/cancers16020324 (PMC10813808; doi:10.3390/cancers16020324)
Supplement: Supplementary file 1 [file cancers-16-00324-s001.zip › cancers-2775915-supplementary.pdf]

Table S1. Correlation between CPET results and cancer treatment.

Variables		Age at diagnosis (years)	Lenght of FU (years)	Total brain RT (Gy)	Lenght ST (months)	Total CB (mg/mq)	Total CIS (mg/mq)	Total IFO (mg/mq)	Total CP (mg/mq)
VO2 picco (ml)	r	0,129	0,22	-0,383	-0,638	-0,567	-0,168	-0,093	0,372
	p	0,836	0,722	0,525	0,247	0,319	0,787	0,882	0,538
VO2 pro kg	r	0,122	0,165	-0,483	-0,703	-0,511	-0,304	-0,23	0,358
	p	0,845	0,791	0,41	0,186	0,379	0,619	0,71	0,554
VO2 %	r	0,141	0,183	-0,464	-0,704	-0,504	-0,256	-0,191	0,336
	p	0,821	0,768	0,431	0,184	0,387	0,677	0,759	0,58
VO2@AT	r	0,132	0,264	-0,244	-0,507	-0,644	0,008	0,092	0,386
	p	0,833	0,667	0,693	0,384	0,24	0,99	0,884	0,521
OUES	r	-0,788	0,651	0,401	-0,272	-0,281	-0,482	-0,387	0,998*
	p	0,113	0,234	0,503	0,659	0,648	0,411	0,52	<0,0001*
OUES%	r	0,086	0,336	-0,21	-0,571	-0,571	-0,015	0,048	0,4
	p	0,89	0,581	0,735	0,315	0,315	0,981	0,938	0,505
VO2/HR	r	0,114	0,287	-0,273	-0,584	-0,584	-0,053	0,016	0,384
	p	0,855	0,64	0,657	0,301	0,301	0,932	0,979	0,524
VO2/HR%	r	0,072	0,27	-0,331	-0,638	-0,564	-0,18	-0,104	0,425
	p	0,909	0,661	0,586	0,247	0,322	0,772	0,867	0,476
DVO2/WR slope	r	-0,073	0,121	-0,299	-0,366	-0,766	-0,331	-0,159	0,627
	p	0,907	0,846	0,625	0,545	0,131	0,587	0,799	0,258
VE/VCO2 slope	r	0,458	-0,424	-0,113	0,269	0,622	0,356	0,201	-0,889*
	p	0,438	0,477	0,856	0,662	0,263	0,557	0,745	0,044*
BR	r	-0,787	0,487	0,882*	0,443	0,15	-0,006	0,013	0,567
	p	0,114	0,405	0,048*	0,455	0,809	0,993	0,984	0,319
FR picco	r	0,469	-0,438	-0,75	-0,456	0,415	-0,331	-0,43	-0,604
	p	0,426	0,46	0,144	0,44	0,487	0,587	0,47	0,281
VE picco	r	0,451	-0,079	-0,728	-0,756	-0,225	-0,209	-0,217	-0,124
	p	0,446	0,9	0,163	0,139	0,716	0,736	0,726	0,843
PCP	r	-0,066	0,363	-0,206	-0,597	-0,58	-0,208	-0,12	0,558
	p	0,916	0,548	0,74	0,287	0,306	0,737	0,848	0,328

AT: Anaerobic Threshold; BR: breathing reserve; CB: carboplatin; CIS: cisplatin; CP: cyclophosphamide; FR: frequency rate; FU: follow up; HR: hearth rate; IFO: ifosfamide; OUES: oxygen uptake efficiency slope; PCP: Peak Circulatory Power; RT: radiation therapy; ST: steroid therapy; VE: ventilation rate; VE/VCO2: minute ventilation/carbon dioxide production; VO2: pulmonary oxygen uptake; WR: work rate.

Table S2. Correlation between metabolic and cardiovasclar biomarkers and cancer treatment.

Variables		Age at diagnosis (years)	Lenght of FU (years)	Total brain RT (Gy)	Lenght ST (months)	Total CB (mg/mq)	Total CIS (mg/mq)	Total IFO (mg/mq)	Total CP (mg/mq)
Lp(a) (mg/dl)	r	0,604	-0,778	-0,975*	-0,152	-0,305	-0,359	-0,261	-0,362
	p	0,28	0,121	0,005*	0,807	0,617	0,553	0,672	0,549
ApoB (mg/dl)	r	0,866	-0,453	-0,289	0,308	-0,449	0,826	0,849	-0,634
	p	0,057	0,443	0,637	0,615	0,449	0,085	0,069	0,251
Leptin (ng/ml)	r	0,35	-0,683	-0,173	0,578	0,381	0,143	0,105	-0,707
	p	0,563	0,203	0,781	0,307	0,527	0,819	0,866	0,182
	r	-0,594	0,261	-0,181	-0,593	0,194	-0,99*	-0,967*	0,593

TNFα (pg/ml)	p	0,291	0,671	0,77	0,292	0,755	0,001*	0,007*	0,292
IL-1β (pg/ml)	r	0,39	0,283	-0,009	-0,424	0,234	0,515	0,346	-0,424
	p	0,516	0,645	0,989	0,477	0,704	0,375	0,569	0,477
IL-10 (pg/ml)	r	0,167	-0,019	0,568	0,767	-0,21	0,891*	0,905*	-0,21
	p	0,788	0,976	0,318	0,13	0,735	0,042*	0,034*	0,735
IL-6 (pg/ml)	r	0,1	-0,518	0,177	0,959*	-0,386	0,32	0,471	-0,081
	p	0,872	0,371	0,776	0,01*	0,522	0,6	0,423	0,897
ET-1 (pg/ml)	r	0,427	-0,547	-0,877	-0,426	0,18	-0,515	-0,534	-0,426
	p	0,473	0,34	0,051	0,474	0,772	0,374	0,354	0,474
AN (μg/ml)	r	0,512	0,097	-0,167	-0,232	-0,5	0,564	0,573	-0,135
	p	0,378	0,877	0,788	0,708	0,391	0,322	0,313	0,829

AN: adiponectin; Apo: apolipoprotein; CB: carboplatin; CIS: cisplatin; CP: cyclophosphamide; ET: endothelin; FU: follow up; IFO: ifosfamide; IL: interleuchin; Lp: lipoprotein; RT: radiation therapy; ST: steroid therapy; TNF: tumor necrosis factor.
